# Intravenous Magnesium Sulphate as an Adjuvant Therapy for Acute Exacerbations of Chronic Obstructive Pulmonary Disease: A Systematic Review and Meta-Analysis

**DOI:** 10.3390/life15060973

**Published:** 2025-06-18

**Authors:** Taimur Farid, Abdousamad Said Omar, Sijah Varar Kandi, Soja Puthiyara Maliyekal, Tze Quan Tuen, Amrutha Thazhuthedath Vijayan, Lakshmi Sudhir Pillai, Ahmed Talaat Deiab, Muhammad Sajid, Ahmad Mesmar, Eman Ibrahim Elzain Hassan, Rijas Keethadath, Hasan Al Chalabi, Tallal Mushtaq Hashmi, Mushood Ahmed, Raheel Ahmed

**Affiliations:** 1 Department of Endocrinology, Torbay and South Devon NHS Foundation Trust, Torquay TQ2 7AA, UK; faridtaimur89@yahoo.com; 2 Department of Medicine, Aneurin Bevan University Health Board, Newport NP19 0BH, UK; 3 Department of Acute Medicine, University Hospitals of Leicester, Leicester LE1 5WW, UK; 4 Department of Respiratory Medicine, Freeman Hospital, Newcastle Tyne NE7 7DN, UK; 5 Department of General Internal Medicine, University Hospitals of Leicester, Leicester LE1 5WW, UK; 6 Department of Cardiology, Sheikh Shakhbout Medical City, Abu Dhabi P.O. Box 11001, United Arab Emirates; 7 Department of Oncology, Huddersfield Royal Infirmary, Huddersfield HD3 3EA, UK; 8 Department of Emergency Medicine, Aneurin Bevan University Health Board, Wales NP20 2UB, UK; 9 Department of Medicine, Rawalpindi Medical University, Rawalpindi 46000, Pakistanmushood07@gmail.com (M.A.); 10 Department of Cardiology, Imperial College London, London SW7 2AZ, UK

**Keywords:** COPD, magnesium sulfate, AECOPD

## Abstract

**Background**: Intravenous magnesium sulfate (IV MgSO_4_) may serve as an effective adjunct therapy to improve clinical outcomes in patients experiencing acute exacerbations of chronic obstructive pulmonary disease (AECOPDs). **Methods**: A comprehensive search was conducted on PubMed, Cochrane, and EMBASE from inception to April 2025 to find eligible studies comparing IV MgSO_4_ plus standard treatment versus standard treatment alone. A random-effects meta-analysis was conducted using RevMan. **Results**: Nine studies (seven RCTs and two observational studies) met the inclusion criteria. Pooled analysis demonstrated that adjunctive IV MgSO_4_ significantly improved peak expiratory flow rate at 45 min (MD = 18.50, 95% CI = 6.36 to 30.65) and significantly reduced hospital admission rates from the emergency department (OR = 0.45, 95% CI = 0.23 to 0.88). No significant differences were observed in the length of hospital stay (MD = −0.83, 95% CI = −2.99 to 1.33) and adverse events (OR = 0.79, 95% CI = 0.20 to 3.13; *p* = 0.73, I^2^ = 25%) between the two groups. **Conclusions**: Adjunct MgSO_4_ in AECOPD is associated with significant improvement in peak expiratory flow rate at 45 min and reduced hospitalization rates. Additional large-scale, multicenter randomized controlled trials are needed to validate and strengthen these findings.

## 1. Introduction

Chronic Obstructive Pulmonary Disease (COPD) ranks among the foremost contributors to global illness and death, with its impact steadily rising due to the aging global population and continued exposure to harmful factors like cigarette smoke and environmental pollutants [1]. COPD is a progressive respiratory condition marked by chronic symptoms and reduced airflow, primarily resulting from structural changes and damage within the airways and alveoli. Over time, this deterioration significantly impairs lung function and contributes to worsening clinical outcomes. The World Health Organization estimates that over 200 million people are affected by COPD globally, with the disease anticipated to become the third leading cause of death by 2030 [2]. This rising trajectory underscores the critical and growing burden of COPD globally, highlighting the urgent necessity for more effective strategies not only in early disease prevention but also in the comprehensive and long-term management of acute exacerbations and disease progression.

The global prevalence of COPD has been increasing, with estimates suggesting that the number of cases among individuals aged 25 years and older will rise by 23% from 2020 to 2050, approaching 600 million cases globally by 2050 [3]. This upward trend is particularly pronounced in low- and middle-income countries, where risk factors such as tobacco smoking, air pollution, and occupational exposures are more prevalent. A key feature of COPD progression is the occurrence of acute exacerbations (AECOPDs), episodes characterized by a sudden worsening of respiratory symptoms that often necessitate urgent medical intervention, hospitalization, and sometimes intensive care support [4]. These exacerbations significantly contribute to the overall disease burden, leading to increased healthcare utilization, reduced quality of life, and higher mortality rates. A study assessing the national inpatient burden of AECOPDs in the United States found that these events are a leading cause of hospitalizations and are associated with substantial healthcare costs [5]. Moreover, frequent exacerbations accelerate the decline in lung function and are linked to increased mortality.

The management of AECOPDs remains a critical aspect of COPD care. Exacerbations of COPD, particularly acute episodes, are triggered by a range of factors, including respiratory infections (both viral and bacterial), air pollution, and non-compliance with prescribed medications. These episodes are not only frequent but also clinically heterogeneous, varying in severity, duration, and underlying pathophysiology. Importantly, the frequency of exacerbations correlates strongly with long-term outcomes, including accelerated decline in lung function, worsening dyspnea, and increased risk of death [3]. Recurrent exacerbations may also lead to irreversible changes in the airways due to persistent inflammation and remodeling, underscoring the need for early identification and intervention. Patients who experience two or more exacerbations per year or one hospitalization for AECOPDs are categorized as “frequent exacerbators”, a phenotype associated with poorer prognoses [6].

Despite the availability of standard treatment options, which typically include short-acting and long-acting bronchodilators (such as beta-agonists and antimuscarinics), systemic and inhaled corticosteroids, antibiotics (in cases with suspected infection), and supplemental oxygen therapy, a substantial proportion of patients continue to experience significant symptom burden and recurrent exacerbations [4]. In this context, there is increasing interest in identifying and evaluating adjunctive therapies that may offer additional benefits beyond the current standard of care. Emerging evidence also indicates a possible role of electrolyte imbalances—particularly hypomagnesemia—in the pathogenesis and management of AECOPDs. Magnesium plays a crucial role in bronchodilation, mucociliary clearance, and modulation of inflammation. Low serum magnesium levels have been associated with increased bronchial hyperreactivity, muscle fatigue, and poor response to β2-agonists [7]. These findings provide a biological rationale for the use of intravenous magnesium sulfate (MgSO_4_) as adjunctive therapy in AECOPD management.

This systematic review and meta-analysis seeks to comprehensively assess the latest findings from both randomized controlled trials and observational studies regarding the effectiveness and safety of intravenous magnesium sulfate when used as an adjunct treatment for acute exacerbations of chronic obstructive pulmonary disease. Through this review, we aim to offer a comprehensive synthesis of evidence that may contribute to the optimization of AECOPD management strategies. Ultimately, our goal is to provide clinicians and healthcare policymakers with robust, evidence-based insights that can support more informed decision-making and improve the clinical outcomes and quality of life for patients experiencing acute COPD exacerbations.

## 2. Methods

This meta-analysis adhered to the guidelines set forth by Preferred Reporting Items for Systematic Reviews and Meta-Analyses (PRISMA), a widely recognized standard for promoting transparency, consistency, and methodological integrity in systematic reviews and meta-analyses [8]. The PRISMA framework provided a structured approach to the identification, screening, eligibility assessment, and inclusion of relevant studies, thereby enhancing the reproducibility and reliability of our findings. To further uphold transparency and reduce the risk of reporting bias, the study protocol was prospectively registered with PROSPERO under the registration ID CRD420251059442. This registration details the review objectives, inclusion criteria, data synthesis methods, and planned subgroup analyses, ensuring a predefined and accountable research methodology. As this study was entirely based on a secondary analysis of data from previously published randomized controlled trials and observational studies, no new data were collected from human participants by the authors. Consequently, formal ethical approval was not required, as the research did not involve any direct interaction with patients or access to personally identifiable information. This meta-analysis adheres to the ethical standards for research using publicly available data and complies with all relevant regulations regarding data usage and reporting.

### 2.1. Data Sources and Searches

An extensive literature review was performed to identify studies assessing the effectiveness and safety of intravenous MgSO_4_ in treating AECOPDs. The search strategy was implemented across several major electronic databases, including the Cochrane Central Register of Controlled Trials (CENTRAL), PubMed, and Embase, covering all records from database inception up to April 2025. In addition to database searches, ongoing and registered clinical trials were also screened through clinical trial registries to ensure the inclusion of unpublished or ongoing studies that met the eligibility criteria. To develop a robust and sensitive search strategy, a combination of Medical Subject Headings (MeSH) and free-text keywords was employed. The terms used included but were not limited to the following: “Chronic Obstructive Pulmonary Disease”, “COPD”, “acute exacerbation of COPD”, “AECOPD”, “magnesium sulfate”, “MgSO_4_”, “randomized controlled trial”, and “RCT”. Boolean operators such as AND and OR were applied to refine the search and combine related terms logically. Moreover, the search strategy was supplemented by manually reviewing the reference lists of all relevant articles and previously published systematic reviews or meta-analyses to identify any additional eligible studies that may not have been captured through database searches alone. The complete search strategy, including all keywords, Boolean operators, and filters applied, is detailed in Table 1. No language restrictions were imposed during the search process to ensure the inclusivity of all potentially relevant data.

### 2.2. Eligibility Criteria

Eligibility for inclusion in this systematic review and meta-analysis was determined using a clearly defined set of inclusion and exclusion criteria, established a priori to uphold methodological integrity and ensure alignment with the study’s objectives. To qualify for inclusion, studies were required to meet the following criteria: (i) Study design: randomized controlled trials or observational studies were included. (ii) Population: Eligible studies enrolled adult patients (aged 18 years or older) presenting with acute exacerbation of chronic obstructive pulmonary disease, regardless of sex or geographic location. (iii) Intervention and comparator: The intervention of interest was intravenous magnesium sulfate administered in conjunction with standard bronchodilator therapy. Comparator groups received standard therapy alone without the addition of MgSO_4_. (iv) Outcomes: Studies were included if they reported at least one relevant clinical outcome, such as peak expiratory flow rate (PEFR), hospital admission rates from the emergency department, length of hospital stay, or adverse events. Exclusion criteria were applied to ensure the internal validity of the findings. Studies were excluded if they met any of the following conditions: (1) quasi-randomized trials, case series, or case reports, which lack the methodological robustness required for meta-analytic synthesis; (2) preclinical studies, including those conducted in animal models, as the objective of this meta-analysis was to assess clinical outcomes in human subjects. This stringent selection process ensured the inclusion of high-quality evidence to evaluate the role of IV magnesium sulfate as adjunctive therapy in the management of AECOPD. Two reviewers independently assessed each study for eligibility, and any disagreements were addressed through consultation with a third reviewer.

### 2.3. Selection Process

We utilized Rayyan, a web-based systematic review tool, to manage and streamline the screening process of the studies identified through our comprehensive database search. Initially, all retrieved citations were imported into Rayyan, which facilitated the automated detection and removal of duplicate records. Following this deduplication process, the remaining studies underwent an initial screening based on their titles and abstracts. This step was performed independently by two authors, each blinded to the other’s decisions, to reduce the risk of bias and ensure a consistent application of the inclusion and exclusion criteria. Subsequently, studies that appeared to meet the eligibility criteria or those that lacked sufficient information for exclusion during the abstract screening phase were subjected to a more detailed full-text review. During this phase, the authors assessed the studies for relevance, methodological quality, and compliance with the predefined eligibility criteria. In instances where discrepancies or disagreements arose regarding the inclusion or exclusion of specific studies, a third author served as an adjudicator. This structured, multi-reviewer approach ensured a transparent and methodologically robust study selection process.

### 2.4. Data Extraction

Data extraction was performed systematically and independently by two reviewers using a standardized, pre-piloted Excel spreadsheet to ensure consistency and accuracy. Key characteristics extracted from each included study comprised the first author’s name, year of publication, study design, country of origin, setting, and sample size. Additional variables included participant demographics such as mean or median age, gender distribution, baseline characteristics, and the specific dosing regimen of intravenous MgSO_4_, including total dosage, rate of infusion, and duration of administration. Details regarding the comparator or standard treatment arm were also documented. The primary outcomes of interest were peak expiratory flow rate (PEFR), and the proportion of participants requiring hospital admission from the emergency department. Secondary outcomes included adverse events and length of hospital stay. Disagreements between the two primary reviewers during the data extraction phase were resolved through adjudication by a third reviewer, ensuring consensus and preserving the objectivity of the selection process.

### 2.5. Risk of Bias Assessment

A thorough quality appraisal was carried out for all included studies to ensure methodological soundness and reduce the likelihood of bias. For randomized controlled trials (RCTs), the Cochrane Risk of Bias tool version 2.0 (RoB 2.0) was utilized. In the case of non-randomized cohort studies, the Risk of Bias in Non-Randomized Studies of Interventions (ROBINS-I) tool was employed. Risk of bias results were visually represented using RoBvis tool for clarity. Disagreements between the two primary reviewers during the bias assessment were resolved through adjudication by a third reviewer, ensuring consensus and preserving the objectivity of the selection process.

### 2.6. Data Analysis

Data synthesis and analysis were conducted using RevMan (The Cochrane Collaboration, Copenhagen, Denmark). A random-effects model was applied to account for potential variability both within and between the included studies, acknowledging that clinical and methodological diversity may influence the observed effects. To evaluate statistical heterogeneity across the included trials, the I^2^ statistic was employed. This metric quantifies the proportion of variability in effect estimates that is due to heterogeneity rather than chance. An I^2^ value exceeding 50% was interpreted as indicative of substantial heterogeneity. When notable heterogeneity was observed, its potential sources were explored through sensitivity analyses. Given the small number of included studies—fewer than ten—it was not statistically appropriate to perform formal assessments of publication bias, such as funnel plots or Egger’s regression test. According to established methodological standards, these tools are unreliable with limited data and may produce misleading results under such conditions. To further investigate the robustness of the findings and identify any study that may disproportionately influence the overall results, a leave-one-out sensitivity analysis was conducted. This approach involves iteratively removing one study at a time and recalculating the pooled estimates, thereby allowing for the identification of outlier studies contributing to heterogeneity or skewing the overall effect size.

## 3. Results

### 3.1. Study Selection

**Figure 1:**
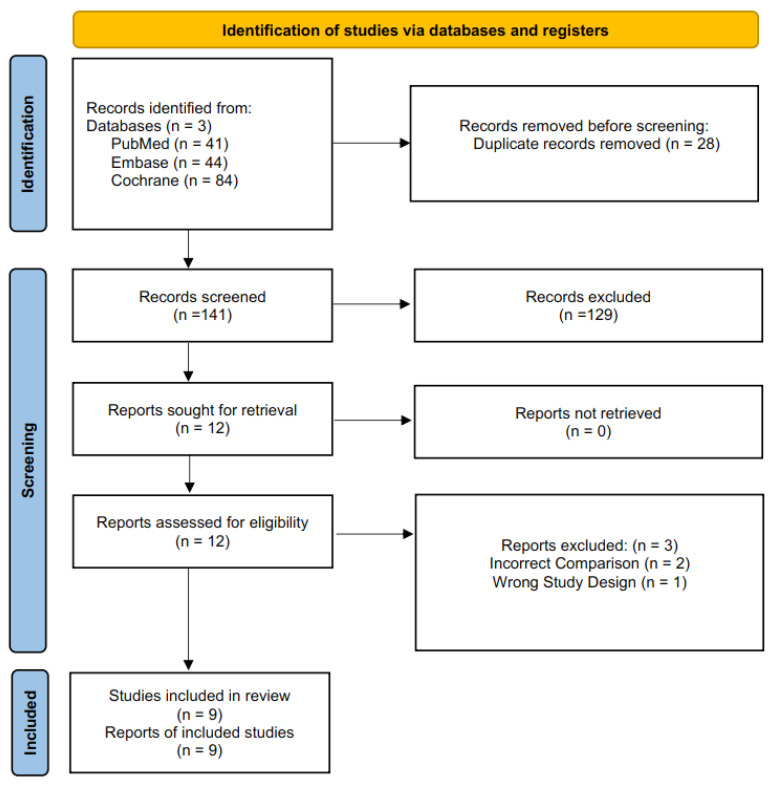
PRISMA flowchart depicting the screening and study selection process.

The initial comprehensive literature search across electronic databases yielded a total of 169 potentially relevant articles. Following the removal of 28 duplicate records, 141 unique studies were identified and screened based on their titles and abstracts. During this initial screening phase, 129 records were excluded for not meeting the predefined eligibility criteria—such as having the wrong study design or target populations, interventions not involving intravenous magnesium sulfate, or outcomes not pertinent to acute exacerbations of chronic obstructive pulmonary disease. Consequently, 12 articles were retained for full-text evaluation to determine their suitability for inclusion in the systematic review and meta-analysis. After a thorough assessment of study design, population characteristics, interventions, and reported outcomes, three studies were excluded due to wrong comparator (2) or ineligible study design (1). Ultimately, nine studies met the inclusion criteria and were included in the final analysis, forming the basis for the quantitative and qualitative synthesis of findings (Figure 1).

### 3.2. Study Characteristics

The included studies were RCTs and observational studies investigating the use of intravenous MgSO_4_ in emergency [9,10,11,12,13,14,15] and inpatient settings [16,17]. Six studies were RCTs conducted in emergency departments involving patients with AECOPD. Two observational studies, conducted in inpatient settings, assessed the effects of MgSO_4_ in a non-randomized fashion. Dosing of MgSO_4_ ranged from 1.2 g to 2.5 g, typically diluted in 100 mL of normal saline and administered over 15–30 min. One study included aerosolized MgSO_4_ (150 mg) in addition to an IV bolus [11]. The mean age of participants ranged from 58.3 to 74.3 years. Detailed study characteristics are presented in Table 2. Most of the trials were judged to have a low risk of bias across all domains. Two trials [10,11] had some concerns due to issues in randomization (Figure S1) and deviations from intended interventions. Among the observational studies, only Aishwarya 2022 [16] had a moderate overall risk of bias due to moderate concerns in confounding and selection of participants (Figure S2).

### 3.3. Primary Outcomes

#### 3.3.1. Peak Expiratory Flow at 45 min

**Figure 2:**
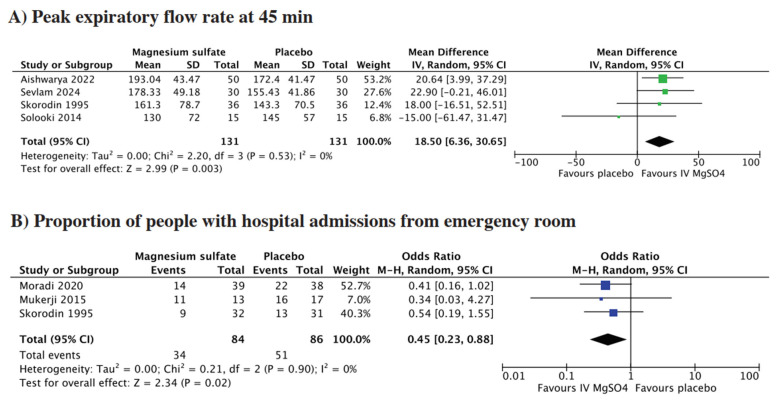
Forest plot for (**A**) peak expiratory flow at 45 min; (**B**) proportion of people with hospital admissions from emergency room [9,10,12,14,15,16].

Four studies reported on peak expiratory flow (PEF) at 45 min. The pooled analysis demonstrated that patients who received adjunct IV MgSO_4_ had a greater PEF at 45 min compared to placebo (MD = 18.50, 95% CI = 6.36 to 30.65; *p* = 0.03, I^2^ = 0%) (Figure 2A).

#### 3.3.2. Proportion of People with Hospital Admissions from Emergency Room

Three studies reported about the proportion of people with hospital admissions from the emergency room. Pooled analysis showed that patients receiving IV MgSO_4_ had significantly lower odds of hospital admission compared to those receiving placebo (OR = 0.45, 95% CI = 0.23 to 0.88; *p* = 0.02, I^2^ = 0%) (Figure 2B).

### 3.4. Secondary Outcomes

#### 3.4.1. Length of Hospital Stay

**Figure 3:**
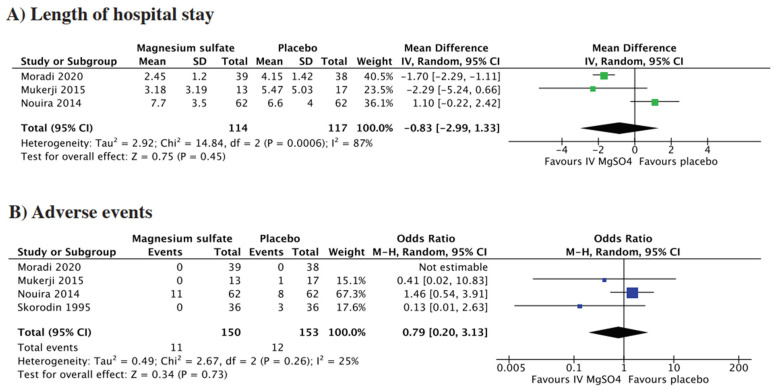
Forest plot for (**A**) length of hospital stay, (**B**) adverse events [9,11,12,14].

Length of hospital stay was reported by three studies. The pooled analysis showed no statistically significant difference between the two groups (MD = −0.83, 95% CI = −2.99 to 1.33; *p* = 0.45, I^2^ = 87%) (Figure 3A). By excluding the study by Nouira et al., sensitivity analysis reduced the I^2^ to 0% and made the finding significant (Figure S3).

#### 3.4.2. Adverse Events

Four studies reported the incidence of adverse events, which included nausea, weakness, dizziness, increased secretions, skin reactions, headache, cardiovascular effects, and tremors. The pooled analysis demonstrated no significant difference between the two groups (OR = 0.79, 95% CI = 0.20 to 3.13; *p* = 0.73, I^2^ = 25%) (Figure 3B).

## 4. Discussion

In this meta-analysis, the addition of intravenous MgSO_4_ to standard bronchodilator therapy was associated with a reduction in inpatient admissions from the emergency department and a notable improvement in peak expiratory flow rate (PEFR) at 45 min compared to standard treatment. These findings reinforce the potential role of MgSO_4_ as an adjunctive treatment in acute exacerbations of chronic obstructive pulmonary disease, highlighting both efficacy and a favorable safety profile. Notably, the length of hospital stay and incidence of adverse events were comparable between groups, suggesting that the addition of MgSO_4_ does not compromise patient safety.

Emerging evidence suggests a potential link between hypomagnesemia and increased susceptibility to AECOPD, highlighting the role of magnesium in respiratory physiology and its therapeutic implications [7]. Magnesium is a vital intracellular cation involved in numerous physiological processes, including enzymatic reactions, neuromuscular conduction, and regulation of smooth muscle tone [18]. These properties underscore its potential utility in managing bronchospastic conditions, such as asthma and COPD.

Magnesium sulfate has been extensively studied as an adjunctive treatment in AECOPD and severe asthma. Its efficacy is supported by prior clinical trials demonstrating that MgSO_4_ enhances bronchodilation, reduces hospitalization rates, and improves lung function when combined with standard therapy [19,20]. The mechanism of action of magnesium sulfate involves antagonizing calcium-mediated smooth muscle contraction, leading to bronchial smooth muscle relaxation [19,21]. Furthermore, MgSO_4_ may enhance β_2_-adrenergic receptor affinity, enhancing the effectiveness and prolonging their duration of action [22]. This is reflected in our findings as the patients who received IV MgSO_4_ had a higher PEFR as compared to those who received standard care only.

Furthermore, our results showed that IV MgSO_4_ therapy also led to a statistically significant reduction in inpatient admission rates. This reduction has important implications for healthcare systems, particularly in low-resource settings or during peak periods when emergency departments and inpatient units are under strain [23]. A treatment that reduces the need for inpatient care while maintaining or improving patient outcomes is of substantial value, both in terms of resource allocation and patient quality of care. The simplicity of administration and low cost of MgSO_4_ further enhance its feasibility as an adjunct therapy in emergency settings. Interventions like MgSO_4_ that optimize acute care outcomes have the potential to significantly reduce the strain on healthcare systems, particularly when they are cost-effective and easily implementable in emergency settings [24]. Current international guidelines, including GOLD [25] and NICE guidelines [26], do not recommend intravenous magnesium sulfate as a routine adjunctive therapy for acute exacerbations of AECOPD. However, in severe or refractory cases, IV MgSO_4_ (typically 1.2–2 g infused over 20–30 min) may be cautiously considered due to its bronchodilatory effects, although supporting evidence remains limited.

The safety profile was reassuring as common adverse effects like nausea, weakness, dizziness, increased secretions, skin reactions, headache, cardiovascular effects, and tremors were comparable between both groups. Notably, the therapeutic window for magnesium is relatively wide, and side effects typically arise only with excessive dosing or in patients with compromised renal function. Sensitivity analysis further elucidated the impact of individual studies on the overall findings. Specifically, excluding the study by Nouira et al. reduced heterogeneity to zero and revealed a statistically significant reduction in hospital stay for patients receiving intravenous MgSO_4_. This suggests that variations in study design, particularly dosing strategies and patient populations, may have influenced the pooled results. Nouira et al.’s study used a different dosing regimen compared to other included studies, which might explain its outlier effect [11]. Understanding such nuances is crucial when interpreting meta-analytic data, as methodological heterogeneity can obscure true treatment effects.

Lastly, the current review has certain limitations. The analysis was based on study-level data rather than individual patient data (IPD), which limits our ability to conduct more detailed subgroup analyses. For instance, we could not assess whether the efficacy of magnesium sulfate varies according to baseline COPD severity, presence of comorbid conditions such as cardiovascular disease or diabetes, or prior history of exacerbations. Such analyses are essential for personalizing treatment and identifying patient groups most likely to benefit from adjunctive magnesium therapy. Furthermore, the included studies varied in several key aspects, including dosing protocols, duration of therapy, criteria for hospital admission, and definitions of outcomes. Such clinical heterogeneity introduces potential confounding factors that may influence the observed treatment effects. While the use of a random-effects model helps account for between-study variability, it does not eliminate the underlying differences in study design. Standardization of future trials in terms of dosing regimens, outcome measures, and follow-up periods would enhance the comparability of results and strengthen the evidence base. The studies varied in their treatment protocols and dosing strategies, which may have influenced the observed outcomes. A formal assessment of publication bias was not possible due to the inclusion of fewer than 10 studies.

Lastly, the long-term impact of adjunctive magnesium therapy on COPD outcomes warrants investigation. While acute improvements in lung function and hospitalization rates are important, understanding whether these translate into better long-term disease control, fewer subsequent exacerbations, and improved quality of life is critical. Incorporating patient-reported outcomes and cost-effectiveness analyses into future trials would also provide a more comprehensive assessment of magnesium’s role in COPD management.

## 5. Conclusions

In patients with acute exacerbation of COPD, adjunctive intravenous magnesium sulfate was associated with a statistically significant improvement in peak expiratory flow at 45 min and a significant reduction in hospital admission rates from the emergency department, with a favorable safety profile. These findings suggest that intravenous MgSO_4_ may serve as an effective adjunct therapy in AECOPD, particularly in settings where rapid symptom resolution and hospital avoidance are critical. However, to establish the definitive clinical benefits of adjunct IV MgSO_4_ in COPD management, well-designed, large-scale, multicenter randomized controlled trials are warranted. These studies should aim to standardize treatment protocols, explore optimal dosing strategies, and assess long-term outcomes, including quality of life and cost-effectiveness.

## Figures and Tables

**Table 1:** Search strategy.

Database	Search String	Records
PubMed/MEDLINE	(“Magnesium Sulfate”[Mesh] OR “magnesium sulfate”[tiab] OR “MgSO_4_”[tiab] OR “magnesium”) AND (“Pulmonary Disease, Chronic Obstructive”[Mesh] OR “chronic obstructive pulmonary disease”[tiab] OR “COPD”[tiab]) AND (“Acute Disease”[Mesh] OR “acute exacerbation”[tiab] OR “acute attack”[tiab] OR “exacerbation”[tiab])	41
Cochrane	(“magnesium sulfate” OR “MgSO_4_” OR magnesium) AND (“chronic obstructive pulmonary disease” OR COPD) AND (“acute disease” OR “acute exacerbation” OR “acute attack” OR exacerbation)	44
Embase	(“magnesium sulfate” OR “MgSO_4_” OR magnesium) (“chronic obstructive pulmonary disease” OR COPD) (“acute disease” OR “acute exacerbation” OR “acute attack” OR exacerbation)	84

**Table 2:** Baseline characteristics of included studies.

Author (Year)	Sample Size		Sex		Current Smoker		Study Design	Setting	Mean Age	Patient Population	Intervention
	MgSO_4_	Placebo	Male	Female	MgSO_4_	Placebo					
Skorodin 1995 [9]	36	36	70	2	NR	NR	RCT	Emergency department	64.7 ± 8.2	Patients with acute COPD exacerbation with COPD duration of 11 years, smoking history of 40 years, and average initial SpO_2_ of 92.1% following albuterol.	IV 1.2 g of MgSO_4_ over 20 min plus standard care.
González 2006 [17]	12	12	24	0	NR	NR	RCT	Inpatient	64 (57–78)	Patients with acute COPD exacerbation, with mean weight of 76 ± 7 kg, and height of 162 ± 13 cm. All patients underwent baseline spirometry and received standard COPD management including bronchodilators, corticosteroids, oxygen, and antibiotics.	A 20 min intravenous infusion of 1.5 g of magnesium sulfate dissolved in 100 mL of 0.9% saline solution.
Solooki 2014 [10]	15	15	21	9	NR	NR	RCT	Emergency department	68.5 ± 9.06	Patients with acute COPD exacerbation, with mean pretreatment FEV1 of 26% ± 12 in the intervention group and 35% ± 18 in the control group.	2 g magnesium sulfate diluted in 100 mL normal saline infused over 20 min was administered
Nouira 2014 [11]	62	62	95	29	58	52	RCT	Emergency department	69.06 ± 8.21	Patients with acute COPD exacerbation. Common comorbidities included arterial hypertension, left heart failure, and diabetes. Participants had a mean COPD duration of ~10–13 years and were mostly current smokers.	150 mg of magnesium sulfate in 4 mL of normal saline via aerosol mask (driven by pressurized air at 10 L/min), along with an intravenous bolus of 1.5 g magnesium sulfate in 10 mL.
Mukerji 2015 [12]	14	19	24	9	4	5	RCT	Emergency department	74.29 ± 10.82	Patients with acute exacerbation of COPD with mean pack years of 40 ± 27.9 years in intervention group and 38.8 ± 18.2 years in control group.	2 g of intravenous magnesium sulfate diluted in 20 mL of 0.9% saline, administered over 15 min.
Jahanian 2021 [13]	30	30	14	46	16	17	RCT	Emergency department	64.35 ± 5.61	Patients with acute exacerbation of COPD presenting to emergency department with similar baseline FEV1 52.56 ± 6.66 in intevention group and 50.90 ± 7 incontrol group.	IV infusion of magnesium sulfate (2 gr in 100 mL of normal saline) over 30 min.
Moradi 2020 [14]	39	38	NR	NR	NR	NR	RCT	Emergency department	NR	Patients with acute exacerbation of COPD with no significant differences in age, sex, vital signs, PEFR, and DSS	2.5 g of MgSO_4_ (5 mL of 50% solution) in 50 mL of normal saline over 15 min.
Aishwarya 2022 [16]	50	50	NR	NR	NR	NR	Observational	Inpatient	NR	Patients with acute exacerbation of COPD with pretreatment PEFR of 171.2 ± 44.03 in intervention group and 168 ± 40.33 in control group.	IV MgSO_4_ 2 g in 100 mL of normal saline slowly over a period of 20–30 min.
Selvam 2024 [15]	30	30	36	24	14	12	Observational	Emergency department	58.33 ± 9.66	Patients with acute exacerbation of COPD had a mean exacerbation frequency of 1.47 ± 1.41 in the MgSO_4_ group and 1.37 ± 1.07 in the placebo group over the past year.	A single dose of intravenous MgSO_4_ infusion 2 g in 100 mL of normal saline slowly infused over a period of 20–30 min

## Supplementary Materials

The following supporting information can be downloaded at: https://www.mdpi.com/article/10.3390/life15060973/s1, Figure S1: Risk of bias for RCTs; Figure S2: Risk of bias for observational studies; Figure S3: Sensitivity analysis for length of hospital stay.
