# Supplementary material for: Intravenous Magnesium Sulphate as an Adjuvant Therapy for Acute Exacerbations of Chronic Obstructive Pulmonary Disease: A Systematic Review and Meta-Analysis
Source: Life (Basel). 2025 Jun 18;15(6):973. doi: 10.3390/life15060973 (PMC12194251; doi:10.3390/life15060973)

Figure S1: Risk of bias for RCTs

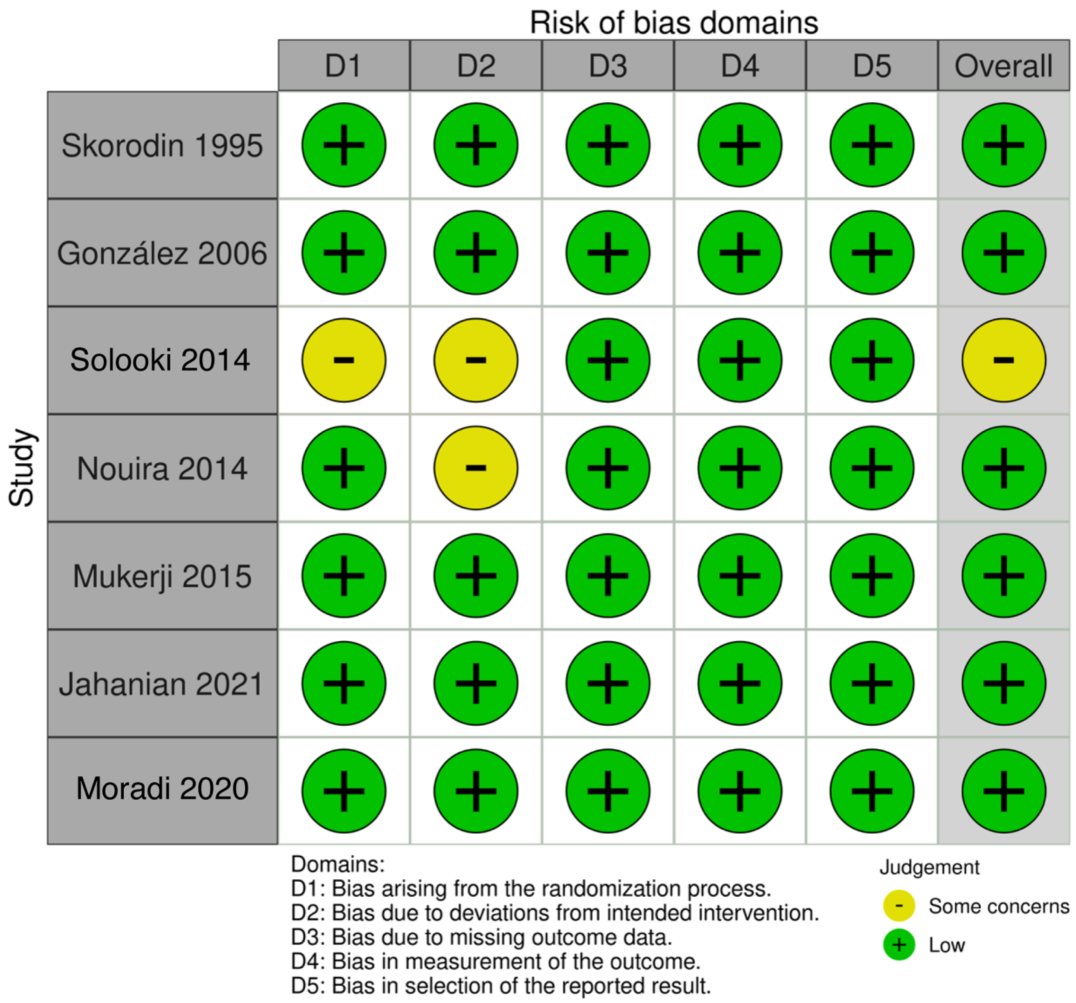

Figure S2: Risk of bias for observational studies

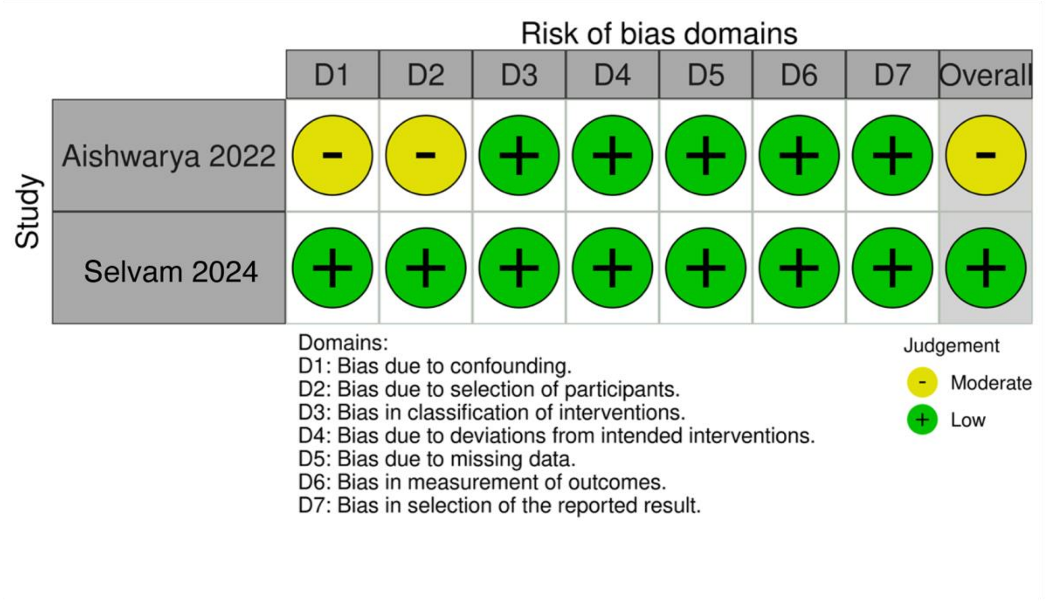

# Figure S3: Sensitivty analysis for length of hospital stay

| | Magnesium sulfate | | | Placebo | | | | Mean Difference |
|---|---|---|---|---|---|---|---|---|
| **Study or Subgroup** | **Mean** | **SD** | **Total** | **Mean** | **SD** | **Total** | **Weight** | **IV, Random, 95% CI** |
| Moradi 2020 | 2.45 | 1.2 | 39 | 4.15 | 1.42 | 38 | 96.2% | −1.70 [−2.29, −1.11] |
| Mukerji 2015 | 3.18 | 3.19 | 13 | 5.47 | 5.03 | 17 | 3.8% | −2.29 [−5.24, 0.66] |
| Nouira 2014 | 7.7 | 3.5 | 62 | 6.6 | 4 | 62 | 0.0% | 1.10 [−0.22, 2.42] |
| **Total (95% CI)** | | | **52** | | | **55** | **100.0%** | **−1.72 [−2.30, −1.15]** |

Heterogeneity: $Tau^2 = 0.00$; $Chi^2 = 0.15$, df = 1 (P = 0.70); $I^2 = 0\%$
Test for overall effect: Z = 5.85 (P < 0.00001)

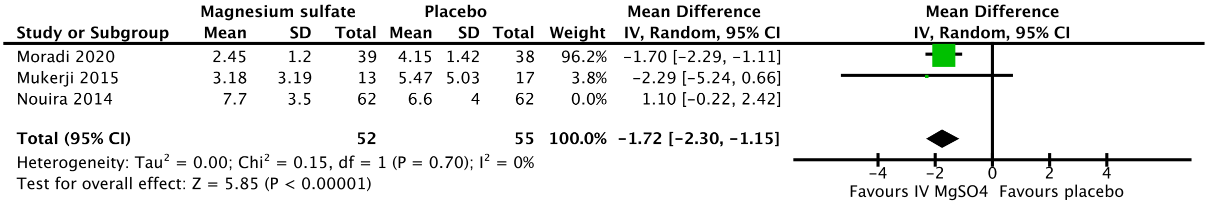

Supplement: Supplementary file 1 [file life-15-00973-s001.zip › life-3695456-supplementary.pdf]
